# One *p*-Factor for All? Exploring the Applicability of Structural Models of Psychopathology within Subgroups of a Population

**DOI:** 10.3390/ijerph18137108

**Published:** 2021-07-02

**Authors:** Darren Haywood, Frank D. Baughman, Barbara A. Mullan, Karen R. Heslop

**Affiliations:** 1 Discipline of Psychology, School of Population Health, Curtin University, GPO BOX U1987, Perth 6845, Australia; frank.baughman@curtin.edu.au (F.D.B.); barbara.mullan@curtin.edu.au (B.A.M.); 2 Health Psychology & Behavioural Medicine Research Group, School of Population Health, Curtin University, GPO BOX U1987, Perth 6845, Australia; 3 WA Cancer Prevention Research Unit, School of Population Health, Curtin University, GPO BOX U1987, Perth 6845, Australia; 4 Curtin School of Nursing, Curtin University, GPO BOX U1987, Perth 6845, Australia; k.heslop@curtin.edu.au

**Keywords:** bifactor, correlated factors, single-factor, *p*-factor, psychopathology, internalising, externalising, thought disorder, subgroups

## Abstract

Structural models of psychopathology have emerged as an alternative to traditional categorical approaches. The bifactor model, which incorporates a general *p*-factor, has become the preferred structure. The *p*-factor is claimed to represent a substantive construct or property of the system; however, recent evidence suggests that it may be without substantive meaning. If a universal substantive *p*, and associated specific factors, is to be developed they not only must be applicable and consistent between populations but also must be applicable and consistent within subgroups of a population. This consistency needs to include not only factor loadings but also factor correlates. We used a simulated data approach to explore the applicability and consistency of four popular models of psychopathology to a range of heterogeneous subgroups and examined the consistency of their neurocognitive correlates. We found that only eight out of sixty-three subgroups fitted any of the models with all significant loadings, no negative loadings, no non-positive-definite identification issues, and no negative variance. All eight of these subgroups fit the correlated factors model, none fit the original bifactor model, four subgroups fit the revised bifactor model, and one subgroup fit the single-factor model. Correlates of the factors also varied substantially between the subgroups fitted to the same model. We discuss the implications of the findings, including the implications for the development of universal substantive factors of psychopathology.

## 1. Introduction

Methodologies using confirmatory factor analytic (CFA) techniques have recently arisen as alternatives to traditional diagnostic approaches in the study of psychopathology. These approaches have risen to mitigate the issues of extensive comorbidity and diagnostic instability found when using traditional categorical systems, such as the *Diagnostic and Statistical Manual* (see [1]). Factor analytical approaches typically take a dimensional approach to the presentations of psychopathology and organise hierarchical structures of specific and more general factors [1]. In essence, these approaches examine the number (and type) of psychological symptoms that are present in a population and then fit these symptoms onto statistical models that posit different structural relations between higher order factors. The bifactor model of psychopathology, comprising of a hierarchical structure of a range of psychopathological symptoms and a smaller collection of higher-order factors, has emerged as the preferred CFA model to summarise psychopathology [1,2,3]. Particularly noteworthy from the bifactor literature are (1) the findings that a single higher-order factor emerges from the statistical analysis of symptoms, and (2) the claim that this factor (subsequently named the *p*-factor; as in ‘psychopathology factor’) may refer to an individual’s propensity towards psychological disease and illness (see [1,4]). Whilst both these points relate to issues that occur when cognitive processes go awry, they are highly similar in nature to claims regarding the structure of the typical development of intelligence. For instance, both the *p*-factor and the *g*-factor (the general factor of intelligence) emerge from the analysis of population data [4]. However, whereas the *g*-factor is assumed to relate to the typical development of intellectual abilities, the *p*-factor is held to represent atypical psychological functioning. Additionally, similar to work on the structure of intelligence is the claim that levels of *p* at the individual level are normally distributed within the population [4].

The key reason why *p* is of interest is the claim that one’s *p* represents a substantive property of the system that determines one’s propensity towards psychopathology [4,5]. In intelligence, *g* has been variously argued to relate to properties such as the capacity of the system, the speed of information processing, or the complexity of information that is represented, e.g., [6]. In the study of the *p*-factor, claims for *p* representing a substantive construct are popular. For example, it has been suggested that *p* may primarily represent functional impairment [7], neuroticism [8], cognitive abilities (see [1]), impulsive responsivity to emotion [9], and disordered thought [5]. However, there is a range of evidence suggesting that, in a purely statistical state, the *p*-factor may not realistically represent a substantive construct or property (e.g., [10]).

The *p*-factor is a function of the dataset from which it is extracted [11,12], and therefore it is inherently fluid and is a different factor depending on the characteristics of the sample and the methods of extraction [13]. Lahey et al. [1] describes *p* as a “weighted-average” (p. 61) of aspects of all the symptoms assessed. Indeed, Fried et al. [3] showed that *p* derived from bifactor models is not notably different to a simple sum of symptoms. Furthermore, even though a bifactor model is often chosen over a correlated factors model (a model with no *p*-factor and correlated specific factors) due to better fit (e.g., [4]), Greene et al. [2] showed that fit statistics unfairly favour the more accommodating bifactor model. These issues have led to multiple authors claiming that if the *p*-factor is to be a substantive construct, substantive *p* must be built “on-top” of statistical *p* [2,3,10]. That is, a theoretical construct of *p* not only must be informed by its statistical make up but also must incorporate predefined predictors and boundaries so that *p* can be falsified [3,10,13]. The development of a universal substantive *p* may have important implications for etiological and treatment domains of psychopathology [1,3]. However, for substantive *p* to be useful in the treatment setting, it must be a construct applicable to every individual in the population. On the individual level, a person’s *p* score is a function of the sample, and therefore would differ depending upon the attributes of the dataset in which they lie. This leaves the question of: Does a population-derived *p* adequately represent the overall symptomology of *each* individual in the sample?

Previously, we claimed that there is a need to assess the applicability and consistency of structural models in heterogeneous subgroups of a population [14]. Bifactor models have been successfully fitted to normative and clinical populations (even though the makeup of *p* changes); however, it is unclear if structural models can fit heterogeneous subgroups *within* a population [14]. It is possible that the factors of psychopathology within a dataset are differentially applicable to a number of individuals in the sample. For example, the *p*-factor might not have substantial utility for a number of individuals or subgroups within a sample, even though the factor has utility for the sample as a whole. Specifically, this may mean factor scores derived from within a subgroup may be better (or worse) at representing those individuals’ symptomologies when compared with factor scores derived from the total sample. However, an alternative model (e.g., a correlated factors model) may be a better fit for some subgroups within a sample or, indeed, no substantial model would adequately account for the subgroups’ symptoms. Practically, this may mean that when parsing out the remaining individuals from a population for a collection of individuals or subgroups within a dataset, the *p*-factor and/or the specific factors of psychopathology might (a) not be applicable, (b) have different factor loading patterns, and/or (c) have different correlates or correlates with different utility. Any of these possibilities would suggest that the factors would have a different substantive meaning within each subgroup when compared with the sample as a whole. However, it is unknown the degree to which subgroups with clear symptomatic boundaries, but with adequate variation to use CFA techniques, could be fit to structural models of psychopathology. This has implications for the representativeness of both statistical *p* and any potential, universal, substantive *p*. Therefore, consideration must also be extended from not just what the *p*-factor might represent *between* samples (e.g., [13]) but also to what it might represent *within* samples.

Levin-Aspenson et al. [13] call for an agreed upon definition of the *p*-factor with corresponding expectations of what constructs should, and should not, correlate with the factor. However, if the *p*-factor has substantial differences in factor loading patterns within a subgroup of a sample, or if a meaningful number of similar individuals within the sample are not adequately represented by the *p*-factor (or the second-order factors), there is unlikely to be a universal (i.e., over the population and different groups) agreement on what the attributions of the *p*-factor (and the specific factors) are. In order to develop a universal substantive meaning of the factors of psychopathology, a within-sample exploration of the factors’ applicability and loading characteristics, as well as the stability of their predictors is needed. However, the issue of poor statistical power arises from fitting CFA models to large subgroups within a sample. Data simulation methodologies not only allow adequate power by having a sample size fit to purpose but also allow the user to control the characteristics of the data. These aspects make simulation methodologies particularly suited to questions related to structural models of psychopathology (e.g., see [2]).

To inform considerations for the use of the factors of psychopathology as both a statistical and substantive construct, in this study we aimed to (a) explore the applicability, (b) loading patterns, and (c) factor associations of various popular structural models of psychopathology to subgroups of a sample using a simulated data approach. The findings of this research may be used to assess the likelihood that consistent, universal, substantive constructs of psychopathology may be developed.

## 2. Materials and Methods

### 2.1. Data Generation

#### 2.1.1. Symptoms

**Figure 1:**
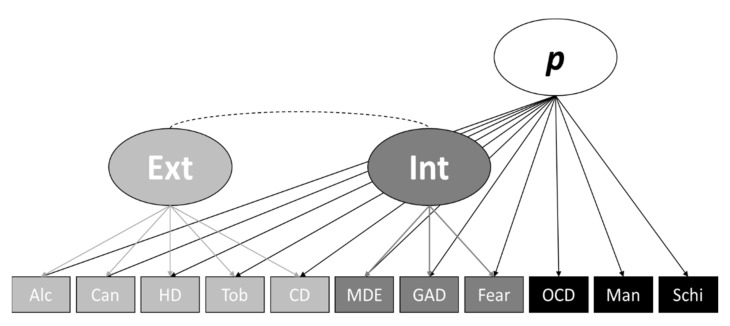
Simulated Data Structural Model. *Note*. Alc = alcohol; Can = cannabis; HD = hard drugs; Tob = tobacco; CD = conduct disorder; MDE = major depressive episode; GAD = generalized anxiety disorder; Fear = fears and phobias; OCD = obsessive-compulsive disorder; Man = mania; Schi = schizophrenia; Ext = externalizing; Int = internalizing.

Caspi et al. [4] used data from the Dunedin Multidisciplinary Health and Development Study (*n* = 1037) approved by the University of Otago Ethics Committee, including symptom counts using the Diagnostic Interview Schedule [15] and a range of potential correlates of psychopathology (see [4] for further details). Similar to Greene et al. [2], we took a top-down data simulation approach whereby the use of existing factor loadings provided by the final bifactor model of Caspi et al. [4] (see Figure 1) allowed us to generate a population dataset consisting of distributions of symptom counts of the 11 disorder categories for 100,000 individual subjects. The distribution of symptom counts were positively skewed (following Greene et al. [2]) to approximately 2.0 across the population. This level of positive skew is representative of distributions of symptoms typically found in the population [16]. The resulting population dataset therefore, represented a summary of symptom counts from Caspi et al. [4].

#### 2.1.2. Symptoms

To explore potential variation in correlates of the factors of psychopathology between samples, we produced IQ scores (analogous to Wechsler Adult Intelligence Scale-IV; WAIS-IV scores) that consisted of normally distributed composite scores (mean = 100, SD = 15) for the Verbal Comprehension (VC), Perceptual Reasoning (PR), Working Memory (WM), and Processing Speed (PS). Each IQ score was generated by matching the associations reported between each subscale score and the externalising factor, internalising factor, and the *p*-factor of Caspi et al. [4]. RStudio was used with the Lavaan package [17] to develop and analyse the simulation data.

### 2.2. Summary of Generated Population Data

Diverging from Greene et al. [2], we did not use Monte Carlo simulations. To better fit the purposes of this research, we produced a single dataset to represent data collected from a single sample as per empirical research and to facilitate a detailed exploration of subgroup characteristics. Our final population dataset comprised of data for 100,000 subjects that included simulated scores on the WAIS-IV subscales, each normally distributed, and the symptoms counts of 11 disorder categories (alcohol, cannabis, hard drugs, tobacco, conduct disorder, fears and phobias, major depressive episode, generalised anxiety disorder, obsessive-compulsive disorder, mania, and schizophrenia), with an average positive skew of approximately 2.. WAIS-IV Scores and symptom counts within the population were simulated from the revised bifactor model and resulting associations from Caspi et al. [4].

**Figure 2:**
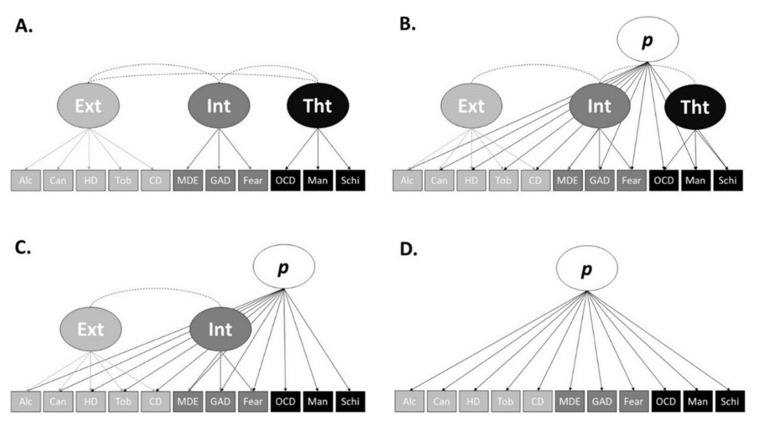
*Note.* Alc = alcohol; Can = cannabis; HD = hard drugs; Tob = tobacco; CD = conduct disorder; MDE = major depressive episode; GAD = generalized anxiety disorder; Fear = fears and phobias; OCD = obsessive-compulsive disorder; Man = mania; Schi = schizophrenia; Ext = externalizing; Int = internalizing; Tht = thought disorder. (**A**) Correlated factors model; (**B**) full bifactor model; (**C**) revised bifactor model; (**D**) single-factor model.

The characteristics of the simulated data closely resembled that of Caspi et al. [4] and had a mean skew of 1.99 (SD = .35) between the 11 observed variables. Table 1 below presents the revised bifactor model factor loadings of Caspi et al. [4], and loadings of the full simulated dataset fitted to the same model are shown in Figure 1. Table 1 also presents bivariate associations between each WAIS-IV subscale and internalising factor, externalising factor, and the *p*-factor for both Caspi et al. [4] and our simulated data (see the Analysis and Subgroup Generation section for details regarding the CFA estimation method and fit indicators used). As shown in Table 1, there is slight divergence between the factor loadings and associations of Caspi et al. [4] and our simulated data. This is due to both random data generation factors, data constraints, potential differences in skew between each Caspi et al. [4] variable and the simulated data, and the use of continuous variables and different estimators (WLSMV vs. MLR). However, the Caspi et al. [4] revised bifactor model of psychopathology fit the simulation data well (χ2(35, *n* = 100,000) = 41.82, CFI = 1.00, TLI = 1.00, SRMR = .002, RMSEA = .001, 90% CI = [.000, .003]), and the simulated data largely retained the factor loading and relationship patterns of Caspi et al. [4]. Furthermore, mirroring Caspi et al. [4], the correlated factors model fit our simulated data well (χ2(41, *n* = 100,000) = 4484.98, CFI = .989, TLI = .986, SRMR = .031, RMSEA = .033, 90% CI = [.032, .034]) (see Table 2 for loading comparisons), and like Caspi et al. [4] the original bifactor model (Figure 2B) had a convergence issue due to the thought disorder factor being subsumed by the *p*-factor. Lastly, like Caspi et al. [4], the single-factor model (Figure 2D) did not fit the data well (χ2(44, *n* = 100,000) = 86,267.82, CFI = .792, TLI = .740, SMRM = .116, RMSEA = .140, 90% CI = [.139, .141]). Therefore, we conclude that our simulated data are a good representation of the data used by Caspi et al. [4]. Following this, the factor loadings were saved back to the dataset, then, as per Caspi et al. [4], we standardised the *p*-factor loadings to a mean of 100 and a standard deviation of 15.

### 2.3. Analysis and Subgroup Generation

#### 2.3.1. CFA

As per Greene et al. [2], we used maximum likelihood estimation (MLR) with robust standard errors and Pearson’s correlations for our CFAs to assess the fit of the models of psychopathology. MLR was chosen for its corrections to chi-square statistics and standard errors when used with skewed data. Sample and estimated variance and covariance differences were assessed via the standardized root-mean-square residual (SRMR; [18]), degree of model fit was determined by root-mean-square error of approximation (RMSEA; [19]), and the comparative fit index (CFI; [20]) and Tucker–Lewis index (TLI) was used to assess fit improvement in relation to a model that is saturated. RMSEA values of <.05 [21] and CFI and TLI values of >.95, with all significant loadings, no negative loadings, no non-positive-definite identification issues, and no negative variance were used as thresholds for a good fit and model utility [18].

#### 2.3.2. Models Tested

Caspi et al. [4] attempted to fit their full sample’s data to four models of psychopathology. A correlated factors model (Figure 2A), a bifactor model (with correlated higher-order factors; Figure 2B), a revised bifactor model (with correlated higher-order factors; Figure 2C) and a single-factor model (Figure 2D). Caspi et al. [4] found the correlated factors model (χ2(1018, *n* = 1000) = 1737.16, CFI = .962, TLI = .958, RMSEA = .027, 90% CI = [.024, .029]), and the revised bifactor model (χ2(1012, *n* = 1000) = 1652.59, CFI = .966, TLI = .963, RMSEA = .025, 90% CI = [.023, .027]) fit the data well. The original bifactor model did not successfully converge (the thought disorder factor was subsumed by the *p*-factor), and the single-factor model did not fit the data well (χ2(1021, *n* = 1000) = 3404.57, CFI = .875, TLI = .862, RMSEA = .048, 90% CI = [.047, .050]). To assess the applicability of each model and the factors of psychopathology *within* a sample, we tested the fit of each of these models on each of our devised subgroups.

#### 2.3.3. Subgroup Determination

To ensure that we captured the heterogeneity and the variation of comorbidity of symptoms within a population, we created overlapping subgroups from our simulated data (i.e., a single case or subject could appear in more than one subsample) determined by thirds of the total sample’s disorder symptom and factor scores. Specifically, each case was characterised as within the lower, middle, or upper third of scores for (1) at least one disorder reflecting a (a) internalising, (b) externalising, or (c) thought disorder, and further by (2) their scores on the (a) externalising and (b) internalising factors and by (3) their *p*-factor score. These permutations resulted in 63 subgroups. Twenty-seven of the subgroups were externalising variants, created using all permutations of upper, middle, and lower scores for at least one externalising disorder, the externalising factor, and *p*. A further 27 were internalising variants, resulting from all permutations of upper, middle, and lower scores for at least one internalising disorder, the internalising factor, and *p*. Lastly, 9 further subgroups were thought disorder variants, resulting from all permutations of upper, middle, and lower scores for at least one thought disorder and *p*. The thought disorder factor did not appear in the Caspi et al. [4] revised bifactor model; thus, thought disorder factor permutations were not possible. In this paper, we refer to subgroups in a A(x)-B(x)-C format, where A represents either the upper (1), middle (2), or lower (3) third of the symptomology of at least one externalising factor, internalising factor, or thought disorder, represented by “x” (“ext”, “int”, or “tht”). B represents either the upper (1), middle (2), or lower (3) third of externalising or internalising factor scores, again with the factor represented by “x”. Lastly, C represents either the upper (1), middle (2), or lower (3) third of *p*-factor scores.

Each of the 63 subgroups were fit to each of the four models of psychopathology to explore the applicability of the sample level structure to the subgroups and the precise factor loading characteristics of each subgroup. Furthermore, bivariate associations between the four WAIS-IV subscales and internalising, externalising, thought disorder, and the *p*-factor in each subgroup were examined.

## 3. Results

Of the 63 subgroups, only 8 fit at least one of the four structural models, with all significant loadings, no negative loadings, no negative variance, and no non-positive-definite identification issues. All eight subgroups fit the correlated factors model (Figure 2A), none fit the original bifactor model (Figure 2B), four subgroups fit the revised bifactor model (Figure 2C), and one subgroup fit the single-factor model (Figure 2D). Of the eight subgroups, four fit only one model, and four fit two of the models of psychopathology. Five externalising and three thought disorder subgroup variants fit the correlated factors model (A), while one externalising and three thought disorder subgroup variants fit the revised bifactor model (C). Lastly, only a single subgroup, an externalising variant, fit the single-factor model. None of the four CFA models of psychopathology fit any of the internalising subgroup variants. The fit indices for the subgroups that fit at least one model are presented in Table 3, and their loadings and associations are presented in Table 4.

### 3.1. Correlated Factors Model (A)

Regarding the correlated factors model (A), symptom loadings on externalising varied substantially between the total sample and the subgroups. Loadings on externalising in the total sample ranged from .545 to .694, while the loadings on externalising among the subgroups ranged from .156 to .666. As expected, externalising variant subgroups had the largest externalising loading differentiation (ranging from .105 to .406), while the thought disorder variants (loadings ranging from .405 to .666) had more similar factor loadings to the total sample. Internalising factor loadings also varied between the total sample and the subgroups. Loadings on internalising for the total sample were typically higher than for the subgroups. The loadings on internalising for the total sample ranged from .563 to .682, while the loadings on internalising for the subgroups ranged from .312 to .556. Loadings of externalising (ranging from .312 to .535) and thought disorder (ranging from .336 to .556) subgroup variants on the internalising factor were similar. Lastly, factor loadings on thought disorder between the full sample and the subgroups varied considerably. Loadings on thought disorder in the total sample ranged from .709 to .968, while the loadings on thought disorder among the subgroups ranged from .109 to .935. Thought disorder variant subgroups (loadings ranging from .109 to .921) and the externalising variants (loadings ranging from .115 to .935) had similar variation in factor loadings on thought disorder. Subgroup variants of higher thought disorder symptoms did not typically load higher than the other subgroups on the thought disorder factor, instead the combination of total sample *p*-factor level and thought disorder symptom level seemed to drive the factors loadings. For example, subgroup 1(tht)-X−1 had thought disorder loadings ranging from .403 to .879, while subgroup 2(tht)-X−2 had thought disorder loadings ranging from .109 to .568. Similarly, externalising variant 1(ext)−1(ext)−3 had higher factor loadings on externalising (ranging from .206 to .307) than subgroup variant 3(ext)−3(ext)−1 (ranging from .156 to .222).

Out of the eight subgroups that fitted the correlated factors model well, four had a negative association between internalising and externalising. This contrasts with the positive association of .296 between the externalising and internalising factors of the full simulation sample. Externalising–internalising association among the four subgroups with a positive relationship ranged from .257 to .800 and included only externalising variant subgroups. The associations among the four subgroups with negative relationships ranged from −.381 to −.050 and included one externalising and three thought disorder variant subgroups and had no consistencies in thirds rankings. This suggests that even though the models with negative relationships between externalising and internalising fit the correlated factors model well, the characteristics of these models are fundamentally different from that of the total sample.

### 3.2. Revised Bifactor Model (C)

Regarding the revised bifactor model (C), one externalising subgroup variant and three thought disorder variants fit the model well, and, like the correlated factors model, symptom loadings on externalising for the revised bifactor model (C) varied substantially between the total sample and the subgroups. Loadings on externalising in the total sample ranged from .368 to .629, while the loadings on externalising among the subgroups ranged from .079 to .662. Loadings on internalising for the subgroups were typically higher than the full sample, ranging from .248 to .459, while the total sample ranged from .247 to .347; however, loadings were closer to the full sample loadings when compared to externalising. This may be expected as no internalising variant subgroups fit the revised bifactor model. The only externalising subgroup variant that fit the revised bifactor model was the subgroup with the lowest externalising loadings (ranging from .079 to .338), even though this subgroup only contained participants in the upper third for at least one externalising disorder, as well as the upper third for the externalising factor score and *p*-factor score (3(ext)/3(ext)/3). The other subgroups that fit the bifactor model well were all thought disorder variants, with matching thirds between their thought disorder symptoms and their *p*-factor scores. This supports the suggestion that in the Caspi et al. [4] revised bifactor model, the *p*-factor largely represents thought disorder [5].

Symptom loadings on the *p*-factor differed substantially between the subgroups and the total sample. Externalising disorder symptom loadings on *p* ranged from .030 to .309 in the subgroups and .294 to .398 in the total sample. Loadings of externalising disorder symptoms on *p* were generally lower than in the total sample, with the 2(tht)-X−2 subgroup providing the lowest externalising disorder loadings on *p* ranging from .030 to .047. Internalising disorder symptom loadings on *p* ranged from .051 to .476 in the subgroups and .476 to .605 in the total sample. Like externalising, loadings of internalising disorder symptoms on *p* were generally lower than in the total sample, and once again the 2(tht)-X−2 subgroup provided the lowest internalising disorder loadings on *p*, ranging from .051 to .092. Thought disorder symptom loadings on *p* once again differed substantially between the subgroups, ranging from .109 to .922, and the total sample, ranging from .709 to .805. Again, the 2(tht)-X−2 subgroup provided the lowest thought disorder loadings, ranging from .109 to .573. The loading characteristics of 2(tht)-X−2 provide an example of a subgroup where, although the bifactor model fit well, the *p*-factor is not representative of the participants’ propensity towards psychopathology, and rather the lower-order factors (i.e., externalising and internalising) are better representations of the subjects’ collective symptoms.

The association between externalising and internalising for all four subgroups that fit the bifactor model well were negative and ranged from −.148 to −.401, compared with the full sample externalising–internalising association of −.387. Only the externalising subgroup variant had the lowest externalising–internalising relationship (−.148) and differed the greatest when compared with the total sample. The thought disorder variants had an externalising–internalising relationship ranging from −.391 to −.401, closely resembling the total sample and providing more evidence that thought disorder drives the *p*-factor in this revised bifactor model.

### 3.3. Single-Factor Model (D)

Only a single subgroup, 2(ext)/1(ext)/3, fit the single-factor model well, indicating *p* alone is generally unable to successfully account for the symptoms of psychopathology at the total sample and the subgroup level. Within subgroup 2(ext)/1(ext)/3, externalising and internalising disorders loaded on *p* at a similar level. Externalising disorders loadings on *p* ranged from .318 to .409, and internalising disorders loadings on *p* ranged from .287 to .421; however, similar to the correlated factors and bifactor models, the thought disorders loaded on *p* to a greater extent. The three thought disorders loadings on *p* ranged from .527 to .915, suggesting that whether second order factors are included (e.g., the bifactor model) or not (e.g., the single-factor model), within the total sample and the subgroups, *p* in this dataset is largely defined by thought disorder symptomology. Given that the single-factor model was not a good fit for the total sample, any comparison of factor loadings between the total sample and the subgroup would not be appropriate.

### 3.4. Factor Associations

The associations between the factor scores derived from within each subgroup and the factor scores for the subgroup derived from the total sample are presented in Table 5. Regarding the correlated factors model (A), the data in Table 5 show that while the subgroup-derived and total-sample-derived factor scores for internalising and thought disorder are very similar (internalising ranging from .921 to .999; thought disorder ranging from .983 to 1.00), subgroup- and total-sample-derived externalising scores vary (ranging from .663 to .999). Externalising factor score associations tended to be lower in the externalising subgroup variants (ranging from .663 to .973) than in the thought disorder subgroup variants (ranging from .997 to .999). This may suggest that externalising factor scores are particularly susceptible to individual and subgroup differences, while internalising and thought disorder scores largely follow the same pattern of scores within the total sample and the subgroups in a correlated factors model. Regarding the revised bifactor model (C), associations between the total-sample- and subgroup-derived factor scores were generally consistent and high (ranging from .941 to 1.00). This suggests the pattern of externalising, internalising, and *p*-factor scores were generally consistent in the total sample and the subgroups. This mirrors the factor loading characteristics that showed, generally, that subgroup *p*-factor loadings followed a similar pattern to the total sample, even when the magnitude of the loadings differed. Similarly, even though the single-factor model did not fit the total sample well, the subgroup-derived *p*-factor for the single subgroup that fit the model well had a very strong relationship with the total-sample-derived *p*-factor (.996).

### 3.5. The Factors of Psychopathology and Intelligence

Next, we compared the total-sample-derived factor scores and subgroup-derived factor scores’ associations with intelligence for each successfully fitted subgroup. The results are presented in Table 6. Regarding the correlated factors model, associations between the total sample factor scores and the WAIS subscales varied greatly from the subgroup *p*-factor scores derived from both the (a) total sample and the (b) subgroups. For example, the associations between the verbal comprehension (VC) subscale and the subgroups’ externalising score derived from the total sample (ranging from −.076 to −.176) and subgroup (ranging from .055 to −.177) varied greatly. Furthermore, only the higher end of that relationship range was representative of the total sample’s externalising relationship with the VC subscale. This may suggest that, within subgroups, factors of psychopathology might be differentially predicted by a range of constructs and thereby have a different substantial meaning for each subgroup. For the correlated factors model, the subgroups’ associations between the WAIS subscales and the total-sample-derived factor scores and subgroup-derived factor scores were generally quite consistent. For example, the subgroup-derived thought disorder factor scores and the total-sample-derived thought disorder factor scores’ associations with the WAIS subscales never differed by more than .004. However, in other instances associations differed between the total-scale-derived and subgroup-derived factors and the WAIS subscales. For example, the relationship between working memory (WM) and the total sample (−.085) and subgroup-derived externalising score (−.135) for subgroup 1(ext)/1(ext)/3 differed by .05.

Regarding the revised bifactor model (C), associations between the total sample factor scores and the WAIS subscales also varied from the subgroup *p*-factor scores derived from both the (a) total sample and the (b) subgroups. For example, the associations between the processing speed (PS) subscale and the subgroups’ *p*-factor score derived from the total sample (ranging from −.030 to −.170) and subgroup (ranging from .029 to −.171) varied greatly compared with the relationship between the total sample’s *p*-factor score and the PS subscale (−.167). However, the subgroups’ associations between the WAIS subscales and the total-sample-derived and subgroup-derived *p*-factor scores were very similar, never differing by more than .003. This suggests that while the characteristics of *p* might differ for each subgroup, as indicated by heterogeneous subgroup associations to the WAIS subscales, *p* derived from within a subgroup and the total sample may reflect the same construct.

Lastly, even although the single-factor model (D) was not a good fit for the total sample and was only a good fit for a single subgroup, associations between the WAIS subscales and the total sample and subgroup-derived *p* scores for the subgroup were very similar. The largest WAIS subscale relationship difference between the total-sample-derived *p* and the subgroup-derived *p* was .005, once again suggesting that within-group-derived *p* is the same construct as total sample *p*.

## 4. Discussion

The main objective of this study was to explore the extent to which popular structural models of psychopathology could be fit to subgroups of individuals within a sample and to explore the similarities and differences between characteristics of the factors within the subgroups. We generated a large sample of symptom and intelligence data that simulated that of empirical work published by Caspi et al. [4] and explored the (a) applicability, (b) loading patterns, and (c) factor associations of four models of psychopathology on a subgroup level.

In analysing the fit of models to each of the 63 subgroups, we were able to determine the extent to which different structural models are successful in capturing the utility of the *p*-factor and the specific factors of psychopathology in representing symptoms. Of the 63 subgroups, each of which was fitted to four different models of psychopathology, only eight were found to fit one or more model well. Put differently, only 3.17% of the models tested showed a reliable fit. At first pass, these results suggest that when exploring the nature of symptoms in subgroups of a population, traditional structural models of psychopathology may be of low utility. This suggestion may seem to contrast with previous research. For example, structural models have been shown to fit well in specific circumstances (i.e., populations with a specific diagnosis), suggesting structural models are robust to mild symptom range limitation [22,23]. However, we explored different combinations of symptoms and factor loadings so as to better capture deviation amongst the general population.

Of the eight subgroups that were found to fit one or more models, all eight fit the correlated factors model, none fit the full bifactor model, only four fit the revised bifactor model, and a single subgroup fit the one-factor model. This may be unexpected, given that bifactor models are more accommodating and fit indices are biased towards bifactor models over correlated factor models [2]. This suggests that within subgroups, including individuals with variability in lower-level symptom counts and higher-level factors scores (derived from the full sample), the *p*-factor is of little utility.

Our results therefore lend support to the suggestion that the *p*-factor is poor at representing variations in the number of symptoms displayed per disorder at the subgroup level, while the specific factors of psychopathology can better account for deviations. Future research should consider these findings when discussing the use of the *p*-factor in individualised treatment settings. Furthermore, it is important to note that the four subgroups fit the revised bifactor model rather than the standard bifactor model. Heinrich et al. [24] explain that *p* in the revised bifactor model, which did not include the specific thought disorder factor, reflects the thought disorder factor and not general psychopathology. When Caspi et al. [4] removed the thought disorder-specific factor from the model, it became an “S−1” bifactor model (see [24,25,26]). The *p*-factor came to represent thought disorder, and internalising and externalising reflected the variance of their indicators over and above the variance subsumed by the thought disorder-referenced *p*-factor [24]. Therefore, as no subgroups fit the standard bifactor model well and only a single subgroup fit the one-factor model well, *p* as understood as a general factor of psychopathology did poorly at accounting for the symptoms of the subgroups. It is also important to acknowledge that the factor loadings and factor covariance for each of the subgroups differed markedly. This variability between different groups mirrors findings of Levin-Aspenson et al. [13] and supports the proposition that the factors of psychopathology reflect different components within different groups. Ultimately, our results suggest that within subgroups of a population, the factors of psychopathology do poorly at accounting for psychopathological symptoms, especially the *p*-factor.

Associations between the total-sample- and subgroup-derived factor scores were generally very high. This suggests that even though the subgroup-derived factors of psychopathology have different loading characteristics when compared with the full sample, for the small number of subgroups that fit a model well, the factors’ scores derived from within the subgroup largely reflect that of the scores derived from the sample. However, there was one notable exception: the externalising factor scores derived from the subgroups for the correlated factors model, in some instances, differed substantially compared with the population derived scores. It is possible that the externalising factor from these models is more tolerant of deviation, while still fitting the assigned CFA model well. Future research should explore what differs between externalising, internalising, and thought disorder to allow for this tolerance.

The associations between the WAIS subscales and both the subgroup- and total-sample-derived factor scores were very similar. This parallels the finding that, generally, the subgroup- and sample-derived factor scores correlated highly for the subgroups. However, the associations between the WAIS subscales and the factor scores of the total sample differed markedly from many of the subgroups’ factor scores. The variation of association strength between the subgroups’ and the full samples’ factor scores and the WAIS subscales has implications for the development of a substantive construct of the factors, including the *p*-factor. For example, if a substantive construct of the *p*-factor was to be developed, with prespecified constructs with which it must correlate within a certain range (e.g., [13]), it is likely this definition of *p* would only be applicable to a full population sample. Therefore, our results suggest that not only do the factors of psychopathology do poorly at accounting for the symptoms of subgroups, especially *p*, but also even for the small number of subgroups for which the factors do have utility, large variations in outcome correlates (in our case WAIS subscales) suggest that developing a universal substantive meaning of the factors would be challenging. Furthermore, these results also reinforce the importance of considering neurocognitive associations with psychopathology at the individual level [14,27].

Recently, authors have described the S−1 bifactor approach to the study of psychopathology as an alternative to the standard bifactor approach [24,25,26]. The S−1 approach allows for the predefinition of the substantive construct of the general factor by loading theoretically important indicators representing a domain of interest directly on the general factor [26,28]. Indeed, the S−1 approach combats many of the issues that we present here with regard to the bifactor model. The approach allows for a clear understanding of what the general factor reflects and for a clear interpretation of what the specific variances, factor loadings, and covariance represent [25]. The S−1 approach is clearly useful at exploring specific questions and the utility of a specific domain of interest in accounting for the common variance of psychopathological symptoms. However, exclusive use of the S−1 approach means abandoning the possibility of a general factor that may reflect the overall propensity towards psychopathology (e.g., [4]). Furthermore, the S−1 approach may not be unaffected by some of the issues we raise here. For example, the correlated factors model also did poorly at accounting for the symptoms of our subgroups, and it also had substantial variability in the strengths of correlates. It is possible that the S−1 approach may be subject to similar issues. Further research should explore these possibilities.

## 5. Limitations and Directions for Future Research

This study, while accounting for the issues of statistical power when conducting this type of research, had some limitations. The simulation approach taken means that the results need to be interpreted with some caution. It is likely that, even although symptom and WAIS data were developed based on known sample characteristics [4], the simulation data differed from the simulated sample. We based symptom distributions on the general skew of symptoms found in the population [16], and this has been successfully utilised in previous research [2]. However, it is likely that the distributions from the base sample varied from our simulated data, which may have implications on the characteristics of the subgroups. Furthermore, we chose to develop a single dataset instead of using Monte Carlo simulations. The use of a single dataset had strengths that were important to this research, such as allowing for a nuanced assessment of the fit, loadings, and associations of each subgroup and the parallel with empirical research. However, Monte Carlo simulations in which the populations conditions may be varied systematically would offer further insight into the boundaries of structural model applicability. We also used continuous variables representing symptom counts. Once again, even although this has been used successfully in previous research [2], it differs from the ordinal data derived from the base sample. Further, the first level of categorisation we used for the subgroups was the symptom level. We used the threshold of at least one disorder representing each factor to be in the lower, middle, or upper third of the total sample. This approach was chosen to account for individuals with just one pervasive difficulty (e.g., pervasive alcohol use while not displaying significant comorbidity). However, this also meant that individuals were often nested within more than one subgroup. Lastly, we tested the four models of psychopathology used by Caspi et al. [4]. These models were chosen based on their popularity in the literature. However, we did not test a higher-order factor model—a model where the specific factors load onto the *p*-factor rather than the *p*-factor taking variance directly from the disorder level (see [29])—or a more traditional bifactor model that does not have correlated specific factors. It is possible that the use of a higher-order factor model or a traditional bifactor model might have produced differential findings.

Future research should look to use Monte Carlo simulations to attempt to validate and extend these findings and also should explore various different grouping methods to accommodate the nesting limitation mentioned above. However, even though simulation and computational methodologies are useful for providing a way to discretely and precisely examine a research question [30], research using large samples of human data is needed to validate these findings. Future research may also measure a larger range of correlates and assess the applicability of the higher-order factor model and the traditional bifactor model at the subgroup level. Research should then explore the development and maintenance of the factor scores on the individual level. This could be done through computational approaches exploring how variations of symptoms over time impact an individual’s factor scores at different time points. Lastly, future research should explore the availability, variability, and correlates of the factors of psychopathology in S−1 bifactor models and should determine if the limitations of the structural models that we present here are applicable to that approach.

## 6. Conclusions

Ultimately, our work suggests that the models of psychopathology we tested are poor at accounting for the symptoms of psychopathology within subgroups of the population. Furthermore, with respect to the utility of the *p*-factor, we showed overwhelmingly that *p* has little utility in accounting for the symptoms of individuals within subgroups. Associations between the WAIS subscales and psychopathology factor scores within the subgroups had significant variability and often differed markedly from the associations in the total sample. Together, these findings not only suggest that may factor models of psychopathology be of limited utility at the subgroup level, and therefore at the individual level, but also suggest that developing universal substantive constructs of the factors may be difficult. If universal substantive constructs of the *p*-factor and the specific factors of psychopathology are to be developed, they may only be useful for describing a sample or population as a whole. The factors may have little utility in describing an individual’s or subgroup’s psychopathological symptom structure. This has implications for the proposed potential utility of the factors within the treatment setting.

## Figures and Tables

**Table 1:** Comparison of Loadings, Covariances, and Associations Between Caspi et al. [4] Revised Bifactor Model in the Original and Simulated Data.

	Loadings/ Associations	Alc	Cann	HD	Tob	CD	Dep	GAD	Fears	OCD	Mania	Schiz	VC~	PR~	WM~	PS~	Ext~ Int
**Caspi et al. (2014)** **(*n* = 1000)**																	
Loadings/associations																	−.471
	*p*	.397	.455	.452	.504	.557	.835	.812	.623	.725	.973	.819	−.129	−.129	−.183	−.176	
	Ext	.626	.811	.709	.420	.691	-	-	-	-	-	-	−.084	−.054	−.028	−.035	
	Int	-	-	-	-	-	.340	.497	.441	-	-	-	.112	.062	−.027	.019	
**Simulation Data** **(*n*= 100,000)**																	
Loadings/associations																	−.387 **
	*p*	.294	.309	.320	.398	.394	.605	.582	.476	.709	.969	.805	−.120 **	−.123 **	−.176 **	−.167 **	
	Ext	.536	.629	.577	.368	.559	-	-	-	-	-	-	−.120 **	−.068 **	−.015 **	−.035 **	
	Int	-	-	-	-	-	.247	.388	.347	-	-	-	.110 **	.060 **	−.007 *	.032 **	

Note. All factor loadings are significant at *p* < .01. Significance of the associations was not reported by Caspi et al. [4] for each variable. * = *p* < .05. ** = *p* < .01. *~ =* association; *df* = degrees of freedom; CFI = comparative fit index; TLI = Tucker–Lewis index; SRMR = standardized root-mean-square residual; RMSEA = root-mean-square error of approximation; Alc = alcohol; Cann = cannabis; HD = hard drugs; CD = conduct disorder; Dep = major depressive episode; GAD = generalized anxiety disorder; Fears = fears and phobias; OCD = obsessive-compulsive disorder; Schiz = schizophrenia; VC = Verbal Comprehension; PR = perceptual reasoning; WM = Working Memory; PS = Processing Speed; Ext = externalizing; Int = internalizing.

**Table 2:** Comparison of Loadings, Covariances, and Associations Between Caspi et al. [4] Correlated Model in the Original and Simulated Data.

	Loadings/ Associations	Alc	Cann	HD	Tob	CD	Dep	GAD	Fears	OCD	Mania	Schiz	VC~	PR~	WM~	PS~	Ext~ Int	Ext~ Tht	Int~ Tht
**Caspi et al. (2014)** **(*n* = 1000)**																			
Loadings/associations																	.328	.577	.849
	Ext	.733	.885	.839	.668	.909	-	-	-	-	-	-	−.139	−.166	−.126	−.126			
	Int	-	-	-	-	-	.972	.934	.704	-	-	-	−.049	−.077	−.154	−.134			
	Tht	-	-	-	-	-	-	-	-	.726	.982	.826	−.115	−.116	−.171	−.166			
**Simulation Data** **(*n*= 100,000)**																			
Loadings/associations																	.296 **	.531 **	.869 **
	Ext	.604	.678	.652	.545	.694	-	-	-	-	-	-	−.166 **	−.125 **	−.114 **	−.125 **			
	Int	-	-	-	-	-	.682	.677	.563	-	-	-	−.075 **	−.094 **	−.166 **	−.148 **			
	Tht	-	-	-	-	-				.709	.968	.805	−.120 **	−.123 **	−.176 **	−.167 **			

Note. All factor loadings significant at *p* < .01. Significance of the associations was not reported by Caspi et al. [4] for each variable. * = *p* < .05. ** = *p* < .01. *~ =* association; *df* = degrees of freedom; CFI = comparative fit index; TLI = Tucker–Lewis index; SRMR = standardized root-mean-square residual; RMSEA = root-mean-square error of approximation; Alc = alcohol; Cann = cannabis; HD = hard drugs; CD = conduct disorder; Dep = major depressive episode; GAD = generalized anxiety disorder; Fears = fears and phobias; OCD = obsessive-compulsive disorder; Schiz = schizophrenia; VC = Verbal Comprehension; WM = Working Memory; PS = Processing Speed; Ext = externalizing; Int = internalizing; Tht = thought disorder.

**Table 3:** Fit Statistics for Subgroups That Fit At Least One Model Well.

Model	Subgroup	Chi-Square	*df*	CFI	TLI	SRMR	RMSEA [90% CI]
**Correlated Factors Model (A)**							
	1(ext)/1(ext)/3 (*n* = 10,684)	133.73	41	.992	.990	.012	.015 [.012, .017]
	3(ext)/3(ext)/1 (*n* = 9,163)	93.22	41	.990	.987	.012	.012 [.009, .015]
	3(ext)/3(ext)/2 (*n* = 11,738)	36.81	41	1.00	1.00	.007	.000 [.000, .005]
	3(ext)/3(ext)/3 (*n* = 12,305)	201.57	41	.990	.987	.015	.018 [.015, .020]
	2(ext)/1(ext)/3 (*n* = 11,306)	122.38	41	.996	.994	.011	.013 [.011, .016]
	1(tht)/X/1(tht) (*n* = 33,218)	525.24	41	.991	.987	.017	.019 [.017, .020]
	2(tht)/X/2(tht) (*n* = 32,610)	52.22	41	1.00	.999	.005	.003 [.000, .005]
	3(tht)/X/3(tht) (*n* = 33,255)	791.28	41	.989	.985	.022	.023 [.022, .025]
**Revised** **Bifactor Model (C)**							
	3(ext)/3(ext)/3 (*n* = 12,305)	35.94	35	1.00	1.00	.005	.001 [.000, .007]
	1(tht)/X/1(tht) (*n* = 33,219)	6.07	35	1.00	.999	.004	.005 [.003, .007]
	2(tht)/X/2(tht) (*n* = 32,610)	24.32	35	1.00	1.00	.003	.000 [.000, .002]
	3(tht)/X/3(tht) (*n* = 33,255)	23.91	35	1.00	1.00	.003	.000 [.000, .001]
**Single-** **Factor Model (D)**							
	2(ext)/1(ext)/3 (*n* = 11,306)	545.76	44	.974	.967	.023	.032 [.019, .034]

Note. *~ =* association; *df* = degrees of freedom; CFI = comparative fit index; TLI = Tucker–Lewis index; SRMR = standardized root-mean-square residual; RMSEA = root-mean-square error of approximation; 90% CI = 90% confidence interval; Ext = externalizing; Int = internalizing; Tht = thought disorder.

**Table 4:** Loadings and Covariances for Subgroups That Fit At Least One Model Well.

Model	Subgroup/ Loadings/ Associations		Alc	Cann	HD	Tob	CD	Dep	GAD	Fears	OCD	Mania	Schiz	Ext~ Int	Ext~ Tht	Int~ Tht
**Correlated Factors Model (A)**																
	1(ext)/1(ext)/3 (*n* = 10,684)															
	Loadings/ associations													.641 **	.955 **	.674 **
		Ext	.206	.255	.234	.259	.307	-	-	-	-	-	-			
		Int	-	-	-	-	-	.506	.495	.377	-	-	-			
		Tht	-	-	-	-	-	-	-	-	.462	.916	.584			
																
	3(ext)/3(ext)/1 (*n* = 9163)															
	Loadings/ associations													.257 **	.655 **	.590 **
		Ext	.156	.161	.182	.209	.222	-	-	-	-	-	-			
		Int	-	-	-	-	-	.438	.467	.426	-	-	-			
		Tht	-	-	-	-	-	-	-	-	.359	.846	.481			
																
	3(ext)/3(ext)/2 (*n* = 11,738)															
	Loadings/ associations													−.116 **	.258 **	.253 **
		Ext	.215	.341	.272	.132	.221	-	-	-	-	-	-			
		Int	-	-	-	-	-	.312	.418	.412	-	-	-			
		Tht	-	-	-	-	-	-	-	-	.115	.512	.196			
																
	3(ext)/3(ext)/3 (*n* = 12,305)															
	Loadings/ associations													.455 **	.733 **	.721 **
		Ext	.290	.374	.351	.307	.415	-	-	-	-	-	-			
		Int	-	-	-	-	-	.527	.535	.401	-	-	-			
		Tht	-	-	-	-	-	-	-	-	.482	.916	.602			
																
	2(ext)/1(ext)/3 (*n* = 11,306)															
	Loadings/ associations													.800 **	.992 **	.732 **
		Ext	.316	.373	.349	.327	.406	-	-	-	-	-	-			
		Int	-	-	-	-	-	.528	.510	386.	-	-	-			
		Tht	-	-	-	-	-	-	-	-	.522	.935	.628			
																
	1(tht)/X/1(tht) (*n* = 33,218)															
	Loadings/ associations													−.135 **	.265 **	.620 **
		Ext	.564	.652	.618	.429	.621	-	-	-	-	-	-			
		Int	-	-	-	-	-	.465	.508	.437	-	-	-			
		Tht	-	-	-	-	-	-	-	-	.403	.879	.525			
																
	2(tht)/X/2(tht) (*n* = 32,610)															
	Loadings/ associations													−.381 **	.062 **	.181 **
		Ext	.553	.663	.605	.405	.603	-	-	-	-	-	-			
		Int	-	-	-	-	-	.336	.466	.395	-	-	-			
		Tht	-	-	-	-	-	-	-	-	.109	.568	.183			
																
	3(tht)/X/3(tht) (*n* = 33,255)															
	Loadings/ associations													−.050 **	.317 **	.710 **
		Ext	.577	.666	.623	.448	.642	-	-	-	-	-	-			
		Int						.536	.556	.430	-	-	-			
		Tht	-	-	-	-	-	-	-	-	.496	.921	.616			
**Revised Bifactor Model (C)**																
	3(ext)/3(ext)/3 (*n* = 12,305)															
	Loadings/ associations													−.148 **		
		*p*	.199	.261	.249	.258	.309	.399	.376	.275	.483	.916	.602			
		Ext	.263	.338	.268	.079	.243	-	-	-	-	-	-			
		Int	-	-	-	-	-	.248	.476	.322	-	-	-			
																
	1(tht)/X/1(tht) (*n* = 33,219)															
	Loadings/ associations													−.391 **		
		*p*	.138	.137	.152	.189	.184	.320	.302	.249	.402	.881	.523			
		Ext	.547	.645	.600	.387	.590	-	-	-	-	-	-			
		Int	-	-	-	-	-	.289	.436	.391	-	-	-			
																
	2(tht)/X/2(tht) (*n* = 32,610)															
	Loadings/ associations													−.399 **		
		*p*	.036	.034	.039	.047	.030	.092	.080	.051	.109	.573	.181			
		Ext	.552	.662	.604	.403	.602	-	-	-	-	-	-			
		Int	-	-	-	-	-	.323	.459	.395	-	-	-			
																
	3(tht)/X/3(tht) (*n* = 33,255)															
	Loadings/ associations													−.401 **		
		*p*	.156	.175	.183	.230	.230	.410	.383	.280	.496	.922	.615			
		Ext	.560	.651	.597	.387	.594	-	-	-	-	-	-			
		Int	-	-	-	-	-	.277	.449	.377	-	-	-			
**Single-Factor Model (D)**																
	2(ext)/1(ext)/3 (*n* = 11,306)															
	Loadings/ associations															
		*p*	.318	.377	.352	.330	.409	.421	.389	.287	.527	.915	.635			

***Note***. All factor loadings significant at *p* < .01. * = *p* < .05. ** = *p* < .01. *~ =* association; *df* = degrees of freedom; CFI = comparative fit index; TLI = Tucker–Lewis index; SRMR = standardized root-mean-square residual; RMSEA = root-mean-square error of approximation; 95%CI = 95% confidence interval; Alc = alcohol; Cann = cannabis; HD = hard drugs; CD = conduct disorder; Dep = major depressive episode; GAD = generalized anxiety disorder; Fears = fears and phobias; OCD = obsessive-compulsive disorder; Schiz = schizophrenia; Ext = externalizing; Int = internalizing; Tht = thought disorder.

**Table 5:** Total Sample and Within Subgroup *p*-factor Associations.

Model	Subgroup		Total Sample EXT	Total Sample INT	Total Sample THT	Total Sample *p*
**Correlated Factors Model (A)**						
	1(ext)/1(ext)/3 (*n* = 10,684)					
		Subgroup EXT	.759 **	.849 **	.992 **	-
		Subgroup INT	.495 **	.996 **	.869 **	-
		Subgroup THT	.723 **	.858 **	.997 **	-
	3(ext)/3(ext)/1 (*n* = 9163)					
		Subgroup EXT	.663 **	.538 **	.924 **	-
		Subgroup INT	.149 **	.976 **	.789 **	-
		Subgroup THT	.348 **	.778 **	.999 **	-
	3(ext)/3(ext)/2 (*n* = 11,738)					
		Subgroup EXT	.973 **	−.220 **	.364 **	-
		Subgroup INT	−.136 **	.921 **	.406 **	-
		Subgroup THT	.279 **	.592 **	.996 **	-
	3(ext)/3(ext)/3 (*n* = 12,305)					
		Subgroup EXT	.887 **	.687 **	.890 **	-
		Subgroup INT	.425 **	.990 **	.887 **	-
		Subgroup THT	.605 **	.888 **	.999 **	-
	2(ext)/1(ext)/3 (*n* = 11,306)					
		Subgroup EXT	.825 **	.908 **	.996 **	-
		Subgroup INT	.691 **	.990 **	.902 **	-
		Subgroup THT	.831 **	.883 **	.997 **	-
	1(tht)/X/1(tht) (*n* = 33,218)					
		Subgroup EXT	.999 **	−.152 **	.343 **	-
		Subgroup INT	−.126 **	.999 **	.771 **	-
		Subgroup THT	.312 **	.764 **	.999 **	-
	2(tht)/X/2 (*n* = 32,610)					
		Subgroup EXT	.997 **	−.454 **	.143 **	-
		Subgroup INT	−.535 **	.949 **	.297 **	-
		Subgroup THT	.136 **	.504 **	.983 **	-
	3(tht)/X/3 (*n* = 33,255)					
		Subgroup EXT	.998 **	.050 **	.368 **	-
		Subgroup INT	.032 **	.998 **	.843 **	-
		Subgroup THT	.413 **	.873 **	1.00 **	-
**Revised Bifactor Model (C)**						
	3(ext)/3(ext)/3 (*n* = 12,305)					
		Subgroup EXT	.986 **	−.501 **	-	.029 **
		Subgroup INT	−.239 **	.941 **	-	.072 **
		Subgroup *p*	.163 **	−.139 **	-	.999 **
	1(tht)/X/1(tht) (*n* = 33,219)					
		Subgroup EXT	.999 **	−.652 **	-	.066 **
		Subgroup INT	−.592 **	.997 **	-	.033 **
		Subgroup *p*	.067 **	.033 **	-	.998 **
	2(tht)/X/2 (*n* = 32,610)					
		Subgroup EXT	.998 **	−.622 **	-	.097 **
		Subgroup INT	−.606 **	.992 **	-	.136 **
		Subgroup *p*	−.020 **	−.032 **	-	.983 **
	3(tht)/X/3 (*n* = 33,255)					
		Subgroup EXT	.997 **	−.587 **	-	.027 **
		Subgroup INT	−.620 **	.998 **	-	.052 **
		*Subgroup p*	−.052 **	.003	-	1.00 **
**Single-Factor Model (D)**						
	2(ext)/1(ext)/3 (*n* = 11,306)					
		Subgroup *p*	-	-	-	.996 **

Note. * = *p* < .05. ** = *p* < .01. VC = Verbal Comprehension; WM = Working Memory; PS = Processing Speed; Ext = externalizing; Int = internalizing; Tht = thought disorder.

**Table 6:** Psychopathology Factors and WAIS Subscale Associations.

Model	Subgroup		VC	PR	WM	PS
**Correlated Factors Model (A)**						
	1(ext)/1(ext)/3 (*n* = 10,684)					
		Total sample EXT	−.083 **	−.080 **	−.085 **	−.109 **
		Total sample INT	−.021 **	−.049 **	−.129 **	−.086 **
		Total sample THT	−.073 **	−.087 **	−.137 **	−.118 **
		Subgroup EXT	−.078 **	−.090 **	−.135 **	−.122 **
		Subgroup INT	−.020 **	−.048 **	−.127 **	−.087 **
		Subgroup THT	−.077 **	−.089 **	−.136 **	−121 **
	3(ext)/3(ext)/1 (*n* = 9163)					
		Total sample EXT	−.076 **	−.043 **	−.014 **	−.046 **
		Total sample INT	.016	−.010	−.010	−.020
		Total sample THT	−.032 **	−.032 **	−.027 *	−.035 **
		Subgroup EXT	−.055 **	−.045 **	−.028 **	−.043 **
		Subgroup INT	.005	−.016	−.010	−.028 **
		Subgroup THT	−.031 **	−.032 **	−.027 *	−.034 **
	3(ext)/3(ext)/2 (*n* = 11,738)					
		Total sample EXT	−.097 **	−.048 **	−.032 **	−.028
		Total sample INT	.028 **	.021 *	−.026 **	−.012
		Total sample THT	−.047 **	−.014	−.041 **	−.043 **
		Subgroup EXT	−.101 **	−.048 **	−.036 **	−.033 *
		Subgroup INT	.031 **	.020 *	−.025 **	−.004
		Subgroup THT	−.050 **	−.015	−.041 **	−.044 **
	3(ext)/3(ext)/3 (*n* = 12,305)					
		Total sample EXT	−.162 **	−.117 **	−.067 **	−.125 **
		Total sample INT	−.055 **	−.071 **	−.125 **	−.139 **
		Total sample THT	−.118 **	−.110 **	−.135 **	−.170 **
		Subgroup EXT	−.157 **	−.127 **	−.114 **	−.167 **
		Subgroup INT	−.062 **	−.075 **	−.122 **	−.139 **
		Subgroup THT	−.121 **	−.111 **	−.135 **	−.171 **
	2(ext)/1(ext)/3 (*n* = 11,306)					
		Total sample EXT	−.099 **	−.101 **	−.116 **	−.133 **
		Total sample INT	−.044 **	−.064 **	−.149 **	−.116 **
		Total sample THT	−.091 **	−.101 **	−.156 **	−.142 **
		Subgroup EXT	−.090 **	−.100 **	−.155 **	−.143 **
		Subgroup INT	−.047 **	−.067 **	−.148 **	−.120 **
		Subgroup THT	−.095 **	−.104 **	−.154 **	−.145 **
	1(tht)/X/1(tht) (*n* = 33,218)					
		Total sample EXT	−.098 **	−.065 **	−.022 **	−.029 **
		Total sample INT	.026 **	.002	−.027 **	−.020
		Total sample THT	−.039 **	−.040 **	−.040 **	−.043 **
		Subgroup EXT	−.099 **	−.065 **	−.023 **	−.039 **
		Subgroup INT	.025 **	.001	−.027 **	−.022 **
		Subgroup THT	−.038 **	−.039 **	−.040 **	−.042 **
	2(tht)/X/2 (*n* = 32,610)					
		Total sample EXT	−.106 **	−.053 **	−.025 **	−.027 **
		Total sample INT	.069 **	.036 **	−.016 **	−.002
		Total sample THT	−.027 **	−.009	−.039 **	−.031 **
		Subgroup EXT	−.106 **	−.054 **	−.024 **	−.026 **
		Subgroup INT	.089 **	.045 **	−.005	.008
		Subgroup THT	−.026 **	−.009	−.037 **	−.029 **
	3(tht)/X/3 (*n* = 33,255)					
		Total sample EXT	−.179 **	−.120 **	−.069 **	−.104 **
		Total sample INT	−.017 **	−.049 **	−.138 **	−.110 **
		Total sample THT	−.102 **	−.103 **	−.153 **	−.150 **
		Subgroup EXT	−.177 **	−.117 **	−.062 **	−.097 **
		Subgroup INT	−.004	−.040 **	−.134 **	−.103 **
		Subgroup THT	−.102 **	−.104 **	−.153 **	−.150 **
**Revised Bifactor Model (C)**						
	3(ext)/3(ext)/3 (*n* = 12,305)					
		Total sample EXT	−.130 **	−.082 **	−.004	−.053 **
		Total sample INT	.122 **	.077 **	.008	.046 **
		Total sample *p*	−.117 **	−.109 **	−.135 **	−.170 **
		Subgroup EXT	−.121 **	−.075 **	.011	−.034 **
		Subgroup INT	.076 **	.043 **	−.017	.003
		Subgroup *p*	−.120 **	−.111 **	−.135 **	−.171 **
	1(tht)/X/1(tht) (*n* = 33,219)					
		Total sample EXT	−.094 **	−.057 **	−.014 **	−.030 **
		Total sample INT	.086 **	.049 **	.005	.016 **
		Total sample *p*	−.039 **	−.041 **	−.040 **	−.043 **
		Subgroup EXT	−.093 **	−.057 **	−.012 *	−.029 **
		Subgroup INT	.082 **	.046 **	.003	.013 **
		Subgroup *p*	−.038 **	−.040 **	−.040 **	−.040 **
	2(tht)/X/2 (*n* = 32,610)					
		Total sample EXT	−.103 **	−.053 **	−.020 **	−.023 **
		Total sample INT	.101 **	.050 **	.006	.017 **
		Total sample *p*	−.027 **	−.009	−.039 **	−.030 **
		Subgroup EXT	−.105 **	−.054 **	−.022 **	−.025 **
		Subgroup INT	.096 **	.048 **	.001	.013 *
		Subgroup *p*	−.027 **	−.009	−.037 **	−.029 **
	3(tht)/X/3 (*n* = 33,255)					
		Total sample EXT	−.145 **	−.080 **	−.002	−.037 **
		Total sample INT	.148 **	.087 **	−.011	.041 **
		Total sample *p*	−.102 **	−.103 **	−.153 **	−.150 **
		Subgroup EXT	−.153 **	−.088 **	−.011 *	−.049 **
		Subgroup INT	.145 **	.083 **	−.018 **	.035 **
		Subgroup *p*	−.102 **	−.104 **	−.153 **	−.150 **
**Single-Factor Model (D)**						
	2(ext)/1(ext)/3 (*n* = 11,306)					
		Total sample *p*	−.087 **	−.098 **	−.156 **	−.141 **
		Subgroup *p*	−.092 **	−.102 **	−.155 **	−.144 **

Note. * = *p* < .05. ** = *p* < .01. VC = verbal comprehension; PR = perceptual reasoning; WM = working memory; PS = processing speed; Ext = externalizing; Int = internalizing; Tht = thought disorder.

## Data Availability

The dataset and Rstudio code will be made available on request to the corresponding author.
